# Diagnostic value of prostate magnetic resonance imaging in men with prostate-specific antigen levels ≥ 15 ng/mL for biopsy decision-making

**DOI:** 10.1186/s13244-026-02235-2

**Published:** 2026-03-02

**Authors:** Samuel Trappe, Lars Schimmöller, Patrick Althoff, Karla Johanna Schero, Sebastian Berg, Jan Philipp Radtke, Irene Esposito, Florian Roghmann, Peter Albers, Gerald Antoch, Rouvier Al-Monajjed, Matthias Boschheidgen

**Affiliations:** 1 Department of Diagnostic and Interventional Radiology, Medical Faculty, University Dusseldorf, Dusseldorf, Germany; 2 Department of Diagnostic, Interventional Radiology and Nuclear Medicine, Marien Hospital Herne, University Hospital of the Ruhr-University Bochum, Herne, Germany; 3 Department of Urology and Neurourology, Marien Hospital Herne, University Hospital of the Ruhr-University Bochum, Herne, Germany; 4 Department of Urology, Medical Faculty, University Dusseldorf, Dusseldorf, Germany; 5 Department of Pathology, Medical Faculty, University Dusseldorf, Dusseldorf, Germany

**Keywords:** Prostate cancer, Multiparametric MRI, PSA value, Risk assessment

## Abstract

**Objectives:**

To determine the value of MRI in men with highly elevated PSA values for the exclusion of clinically significant prostate cancer (csPC).

**Materials and methods:**

In this retrospective bicenter cohort study, consecutive men with PSA values ≥ 15 ng/mL and multiparametric (mp) MRI were included. We excluded patients with acute prostatitis and patients without histopathology or follow-up. Examinations were evaluated regarding MRI quality, PSAD, and PI-RADS classification. For all patients with subsequent biopsy, PC and csPC detection rates were determined. In a subgroup analysis, patients with and without the presence of csPC were compared regarding clinical and MRI parameters.

**Results:**

Finally, 376 patients (median PSA 20 ng/mL) were included. MRI quality was excellent (median PI-QUAL 3). 26% of the patients revealed an MRI with a PI-RADS category 2, 16% were classified as category 3, 12% PI-RADS 4, and 46% showed a PI-RADS 5. A total of 280 patients underwent systematic screening with or without targeted prostate biopsy. Among these, 42% with PSA values ranging from 15 to 116 ng/mL (median 19.5 ng/mL) showed no presence of PC. Overall, csPC detection rates were 94% for PI-RADS 5 and 51% for PI-RADS 4. No csPC were identified in PI-RADS 2, and 8% in PI-RADS 3. Comparative analysis between patients with and without csPC revealed significant differences in age, PSA, PSAD, and PI-RADS (*p* ≤ 0.05).

**Conclusions:**

mpMRI demonstrated excellent performance in the detection of csPC in this high-risk cohort with PSA levels ≥ 15 ng/mL. High-quality MRI helps to exclude csPC in cases with significantly elevated PSA levels to avoid unnecessary prostate biopsies.

**Critical relevance statement:**

mpMRI demonstrated a high diagnostic accuracy for csPCs in men with PSA ≥ 15 ng/mL, and in cases of non-suspicious MRI findings, it can avoid unnecessary biopsies in these patients at risk.

**Key Points:**

- MpMRI demonstrated high diagnostic accuracy in men with PSA values of ≥ 15 ng/mL.
- MpMRI enables the reliable exclusion of csPC in cases with non-suspicious MRI findings in these patients.
- In patients with significantly elevated PSA levels, mpMRI provides an effective risk stratification to avoid unnecessary biopsies.

**Graphical Abstract:**

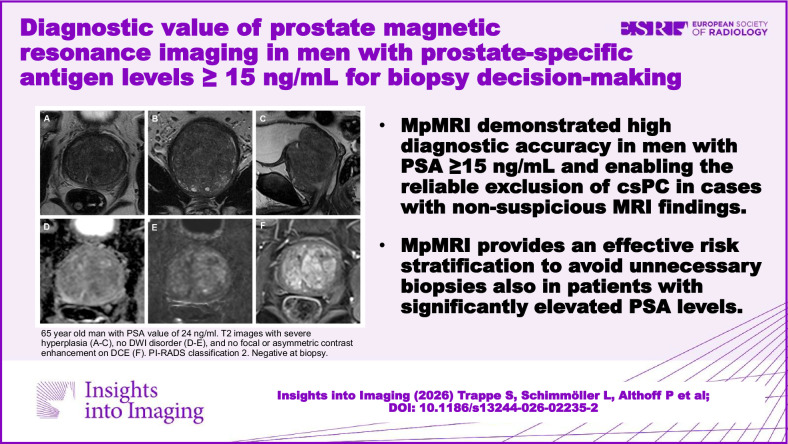

## Introduction

Prostate-specific antigen (PSA) as an oncologic biomarker remains the primary test in suspicion of prostate cancer (PC) [1]. Values tend to increase with lesion size and malignancy. Cut-off values are arguable and still a matter of discussion. Owing to the modest sensitivity of PSA, international guidelines have been unable to provide a threshold recommendation, as clinically significant prostate cancer (csPC) exists at all levels of serum PSA [1, 2]. PSA also lacks specificity, since age, benign prostate hyperplasia, infection, inflammation, or certain medications can all lead to increased serum PSA levels, leading to potential overdiagnosis and unnecessary biopsies [3–5]. Nonetheless, PSA cut-offs, e.g., of 10 ng/mL were used for high-risk classifications [1] or 15 ng/mL for exclusion of Active Surveillance (AS) patients [6]. Multiparametric magnetic resonance imaging (mpMRI) has emerged as a major tool in the decision-making process regarding prostate biopsies [7–11]. Risk assessment using MRI prior to biopsy (MRI pathway), combined with MRI-targeted biopsy, has demonstrated superior diagnostic accuracy compared to the standard transrectal ultrasound (TRUS)-guided approach [9, 11, 12]. Negative predictive value for csPC is excellent, especially in an experienced setting with qualitative MRI, resulting in fewer biopsies and reducing overdiagnosis [5–7, 13, 14]. Most PC missed by MRI are low-risk PC (International Society of Urological Pathology (ISUP) grade group 1) and organ-confined. But, regardless of current guidelines, men with very high PSA values higher than 15 ng/mL are still systematically biopsied in many places.

Data on the diagnostic accuracy and negative predictive value of mpMRI in patients with highly or very highly elevated serum PSA levels are limited. This study aims to analyze the diagnostic performance of MRI in patients with significantly elevated PSA levels to strengthen the role in clinical prebiopsy decision-making and whether they all require biopsy or not. We selected a PSA threshold of ≥ 15 ng/mL to define a high-risk cohort. This cut-off lies in the upper part of the well-established intermediate-risk PSA range (10–20 ng/mL) used in widely adopted risk stratification systems and clinical guidelines.

## Materials and methods

### Study design

The study was approved by the local ethics committee, and written informed patient consent was obtained (Medical Faculty of the Heinrich-Heine-University Düsseldorf; study number/ID: 5910R/2017034171). In this two-center study (University Hospital Düsseldorf, Center A and Marien Hospital Herne, University Hospital of the Ruhr-University Bochum, Center B), consecutive patients with MRI and PSA values ≥ 15 ng/mL were retrospectively included from February 2019 to September 2024 (Center A) and May 2023 to September 2024 (Center B). Both populations originate from university hospitals in Germany. MRI indication was PC suspicion. The PSA checks were carried out as part of an opportunistic screening conducted by general practitioners and urologists. Patients with symptomatic or suspected prostatitis and patients without histopathology or incomplete follow-up were excluded. Clinical information contained age, PSA, prostate-specific antigen density (PSAD), and PI-RADS. For patients who underwent subsequent prostate biopsy, the PC detection rates in the respective PI-RADS categories and the diagnostic performance were analyzed. In addition, a subgroup analysis was performed to compare clinical and MRI parameters between patients with and without csPC. Histopathology was defined as the standard of reference. For patients without biopsy, we assessed 12 months of follow-up with MRI imaging and PSA controls. The primary endpoint was the negative predictive value (NPV) of the PI-RADS classification to exclude csPC in men with high PSA values. Secondary endpoints included comparisons between patients with csPC and those with either no PC or non-significant prostate cancer (nsPC), based on clinical variables such as age, PSA, PSAD, and PI-RADS score.

### Imaging acquisition and biopsy

MpMRI examinations at Center A were performed at a 3 T MRI scanner (Prisma, Siemens Healthcare GmbH) using a 60-channel phased-array surface coil (anterior and posterior part integrate 30 elements each). Examinations at Center B were performed at a 3-T MRI scanner (Vida, Siemens Healthcare GmbH, or Ingenia, Philips) using a 16-/18-channel phased-array surface coil combined with a spine coil (12-/18-channel). MR imaging parameters were chosen according to international recommendations and contained T2-weighted sequences in 3 planes (T2-weighted imaging; turbo spin echo, TSE; axial: 3.0 mm; FOV 130-160 mm), diffusion-weighted imaging (DWI; ss-EPI and/or rs-EPI; 3.0 mm; *b* values 0, 500, 1000 s/mm^2^ plus ≥ calculated 1800 s/mm^2^), and dynamic contrast-enhanced (DCE) imaging (DCE; T1 vibe; 3.0 mm, scan time 3 min, temporal resolution 7–9 s). Apparent diffusion coefficient (ADC) parameter maps were calculated by the scanner using the standard monoexponential model (including b0). MRI/TRUS-fusion biopsy was conducted with a maximum of 3 index lesions and 2 targeted biopsy (TB) cores per lesion and 12-18 systematic biopsy (SB) cores (depending on the size of the prostate), and transrectal or transperineal routing using dedicated biopsy software (e.g., UroNAV, Invivo, or Artemis, Siemens Healthcare) with elastic fusion.

### Image analysis

Image analyses were performed by two experienced radiologists (> 5 years in reading prostate MRI) according to PI-RADS v2.1, meaning standardized lesion evaluation based on T2-weighted, DWI, and DCE imaging. All scans were re-read for the study, and readers were blinded to clinical and histopathologic results. Volumetric analysis and segmentation of the prostate gland were done using specific software (DynaCAD, Invivo, or ProFuse). Additionally, all images were rated according to the prostate imaging quality scoring system (PI-QUAL) scoring system v2, specifically for this study. Biopsy specimens were evaluated by local pathologists and subsequently classified into International Society of Urological Pathology Grade Groups (ISUP GG). ISUP GG ≥ 2 was considered clinically significant.

### Statistical analysis

Statistics were performed using IBM SPSS® Statistics (version 27, IBM Deutschland GmbH). *p*-values < 0.05 were defined as statistically significant. To reduce the number of statistically significant values without clinical relevance, Bonferroni’s *p*-values were corrected for multiple comparisons. Descriptive statistics included mean values and standard deviation for normally distributed variables and median and interquartile range for non-parametric data. Cancer detection rates were calculated for both, PC and csPC. For comparison between csPC and non-significant PC, we applied Welch’s *t*-test to check for significant differences in terms of clinical parameters.

## Results

### Baseline characteristics

Overall, 376 patients from the two centers (Center A and B) were included in this study with a median age of 67 years (IQR 63–75 years). PSA values were equal to or above 15 ng/mL and varied widely up to 620 ng/mL (Table 1). Biopsy and histopathology data of the final biopsy were available for 280 patients, including 207 cases of targeted plus systematic fusion-guided biopsy and 73 cases with SB (Table 2). In 96 patients, no biopsy data were available (e.g., refused or no in-house biopsy), but during a minimum follow-up period of 12 months, none of these patients showed an upgrade in PI-RADS category at follow-up MRI or a suspicious increase in PSA levels. Scan quality was excellent for all included mpMRI with a median PIQUAL v2 score of 3. No scans with insufficient image quality (PI-QUAL score 1) were included.

**Table 1:** Baseline characteristics of the study cohort

		Center A	Center B
Number of patients		208	168
Time period		02/2019 to 10/2023	05/2023 to 09/2024
PSA [ng/mL]	mean ± SD (range)	32 ± 51 (15–620)	27 ± 18 (15–122)
	Median (IQR)	21 (17–30)	20 (17–27)
PSAD [ng/mL/cm^3^]	mean ± SD (range)	0.68 ± 1.90 (0.06–25.83)	0.48 ± 0.47 (0.06–2.54)
	Median (IQR)	0.40 (0.21–0.63)	0.31 (0.18–0.57)
PI-RADS 1–2		44 (21%)	53 (32%)
PI-RADS 3		39 (19%)	23 (14%)
PI-RADS 4		28 (13%)	17 (10%)
PI-RADS 5		97 (47%)	75 (45%)

*PI-RADS* prostate imaging reporting and data system, *PSA* prostate-specific antigen, *PSAD* prostate-specific antigen density

**Table 2:** Characteristics of the biopsy cohort

		Center A	Center B
Number of patients		144	136
PSA [ng/mL]	Mean ± SD (range)	32 ± 58 (15–620)	27 ± 18 (15–122)
	Median (IQR)	20 (17–27)	20 (17–28)
PSAD [ng/mL/cm^3^]	Mean ± SD (range)	0.54 ± 0.74 (0.08–5.9)	0.51 ± 0.50 (0.06–2.5)
	Median (IQR)	0.36 (0.21–0.61)	0.34 (0.18–0.63)
PI-RADS 1–2		33 (23%)	36 (26%)
PI-RADS 3		25 (17%)	20 (15%)
PI-RADS 4		21 (15%)	17 (13%)
PI-RADS 5		65 (45%)	63 (46%)
Negative biopsy		63 (44%)	56 (41%)
ISUP1		6 (4%)	10 (7%)
ISUP2		13 (9%)	17 (13%)
ISUP 3-5		62 (43%)	53 (39%)

*PI-RADS* prostate imaging reporting and data system, *PSA* prostate-specific antigen, *PSAD* prostate-specific antigen density

### PC detection rates and MRI accuracy

**Fig. 1:**
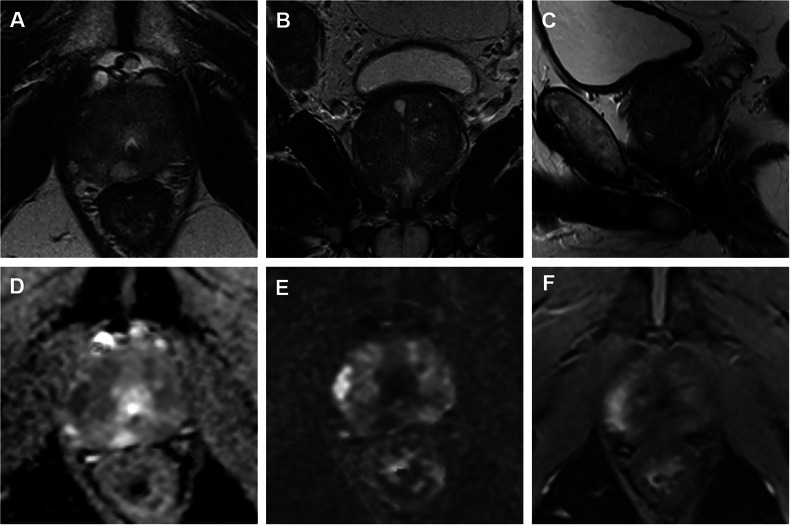
Fifty-nine-year-old men with a PSA value of 135 ng/mL. Diffuse changes on T2 images (**A**–**C**), focal DWI disorder on the right side, anterior and posterior lateral apical in the PZ (**D**, **E**), and focal contrast enhancement on DCE (**F**). On the right side, parailiacal pathological lymph nodes (**B**). PI-RADS classification 5, MR-staging: cT3a cN1. Histopathological revealed an ISUP GG 5 PCA

**Fig. 2:**
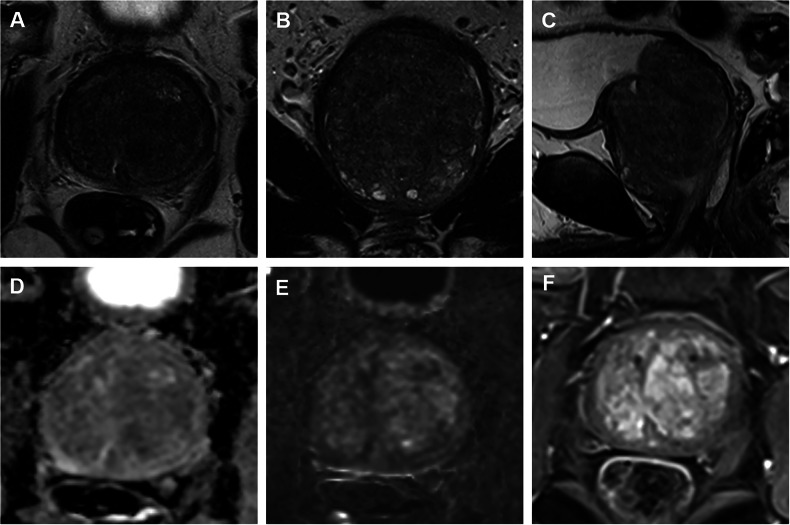
Sixty-five-year-old man with a PSA value of 24 ng/mL. Diffuse changes on T2 images with severe hyperplasia (**A**–**C**), no DWI disorder (**D**, **E**), and no focal or asymmetric contrast enhancement on DCE (**F**). PI-RADS classification 2. Negative at SB

Distribution of PI-RADS classification and biopsy results among the 280 patients with biopsy and histopathological results (either SB or SB + TB) are shown in Table 2. PC was found in 161/280 patients (58%), and 145/280 (52%) of them had csPC (ISUP ≥ 2). PC detection rates for PI-RADS 5 were 98%, with a detection rate of csPC of 94%. Prostate imaging and reporting archiving data system 2 cases showed in 4% a PC and in no cases a csPC (Table 3). Considering the performance of MRI at the different cut-off values (PI-RADS > 3 vs PI-RADS > 4), we observed NPV > 96% (95% CI: 0.88–0.98) for all tests performed and sensitivity of 100% (95% CI: 0.97–1.0) when defining PI-RADS 3 as positive (Table 4). Examples of positive PI-RADS 5 and negative PI-RADS 2 cases are shown in Figs. 1 and 2.

**Table 3:** Cross table with PC detection rates of MRI and systematic and/or targeted biopsy

	Negative	ISUP 1	ISUP 2	ISUP 3-5	Total
PI-RADS 1-2	66 (96%)	3 (4%)	0	0	69
PI-RADS 3	37 (82%)	4 (9%)	3 (7%)	1 (2%)	45
PI-RADS 4	14 (36%)	5 (15%)	6 (15%)	13 (34%)	38
PI-RADS 5	2 (2%)	5 (4%)	20 (15%)	101 (79%)	128
Total	119	16	30	115	280

*PI-RADS* prostate imaging reporting and data system, *ISUP* International Society of Urological Pathology

**Table 4:** Accuracy of MRI for csPC detection with different cut-offs

Cut-offs	ISUP ≥ 2		ISUP ≥ 3
	PI-RADS ≥ 3	PI-RADS ≥ 4	PI-RADS ≥ 4
SEN (95% CI)	100% (0.91–1.0)	97% (0.93–0.98)	99% (0.97–1.0)
SPC (95% CI)	50% (0.38–0.58)	80% (0.73–0.84)	67% (0.63–0.74)
NPV (95% CI)	100% (0.91–1.0)	96% (0.88–0.98)	99% (0.97–0.99)
PPV (95% CI)	67% (0.62–0.75)	83% (0.77–0.88)	67% (0.62–0.71)
ACC (95% CI)	75% (0.68–0.79)	89% (0.84–0.93)	80% (0.75–0.86)

SEN sensitivity, SPC specificity, NPV negative predictive value, PPV positive predictive value, AC accuracy

### Differentiation of csPC

Patients with csPC showed significant differences in key diagnostic parameters (age, PSA. PSAD) compared to those with non-significant PC (nsPC). Patients diagnosed with csPC were generally older (median age 71 vs 67 years; *p* < 0.001), had significantly higher PSA values (23 vs 18 ng/mL; *p* = 0.006), and PSAD (0.57 vs 0.22 ng/mL/cm^3^; *p* < 0.001) (Table 5). Furthermore, both groups differed significantly in PI-RADS categories (median PI-RADS 3 for nsPC vs median PI-RADS 5 for csPC; *p* < 0.001) as all of the csPC were rated PI-RADS 3 or above. In addition, only 4 cases with PI-RADS 3 resulted in a csPC, and 28 cases with PI-RADS ≥ 4 revealed non-significant PC (Table 5). 43% of the ISUP 1 PC occurred in PI-RADS ≥ 3 cases.

**Table 5:** Comparison of no/nsPC and csPC

	Negative or ISUP GG 1	ISUP GG 2-5	*p*-value
Age years (median; IQR)	67 (62–74)	71 (65–76)	< 0.001
PSA ng/mL (median; IQR)	18 (16–22)	23 (19–38)	0.006
PSAD ng/mL/mL (median; IQR)	0.22 (0.15–0.33)	0.57 (0.36–0.91)	< 0.001
PI-RADS (median; IQR)	3 (2–3)	5 (5–5)	< 0.001
PI-RADS ≤ 2	69	0	
PI-RADS ≤ 3	110	4	
PI-RADS ≥ 3	69	142	
PI-RADS ≥ 4	28	138	

*PI-RADS* prostate imaging reporting and data system, *ISUP GG* International Society of Urological Pathology Grade Group, *PSA* prostate specific antigen, *PSAD* prostate specific antigen density

## Discussion

Multiple studies have demonstrated the high sensitivity of mpMRI for detecting csPC [4, 5, 9, 13–15]. However, its performance in patients with markedly elevated PSA levels remains a diagnostic challenge, particularly in determining the indication for biopsy. While elevated PSA levels increase the likelihood of PC, they lack specificity and frequently result in unnecessary biopsies and overdiagnosis [16, 17]. Nonetheless, absolute PSA cut-off levels are part of conventional prediction models and risk assessment systems like d‘ Amico, NCCN, and even Cambridge Prognostic Groups. Our findings indicate that mpMRI reliably excluded malignancy even in patients with PSA levels ≥ 15 ng/mL, while maintaining high sensitivity for detecting csPC. This puts absolute PSA cut-offs in clinical staging systems into question. Although overall diagnostic accuracy was comparable, the prevalence of PI-RADS 5 lesions in patients with PSA levels ≥ 15 ng/mL was approximately threefold higher compared to standard patients undergoing mpMRI (median PSA approx. 8–9 ng/mL). Importantly, csPC was (very) rare in PI-RADS ≤ 3 cases, further reinforcing the high NPV of mpMRI. These findings align with existing literature and support the MRI pathway, in which mpMRI serves as a first-line risk assessment tool and guides biopsy decisions based on imaging results rather than routinely performing systematic biopsies [18–20]. This was also emphasized in the current national German S3 guidelines on PC, with the recommendation not to perform a biopsy in the case of a PI-RADS score of ≤ 2 and low risk PI-RADS score of 3 [6].

The quality of imaging plays a critical role in this diagnostic strategy. High-quality MRI and experienced evaluation directly impact cancer detection and diagnostic confidence [5, 8, 21, 22]. To improve consistency and comparability, Giganti et al introduced the PI-QUAL scoring system for prostate MRI quality assessment [23, 24]. In our cohort, the median PI-QUAL score was 3, indicating excellent imaging quality and fulfilling key prerequisites for reliable interpretation. Based on our results, unnecessary biopsies could have been avoided in 42% of patients (PI-RADS ≤ 3) without compromising csPC detection. This supports the growing evidence that mpMRI can reduce biopsy rates and avoid overdiagnosis of indolent cancers [4, 14]. Even in cases where biopsy is recommended by current guidelines, PI-RADS 3 lesions in patients with PSA ≥ 15 ng/mL should not automatically prompt invasive diagnostics, as we observed only a few csPC in these patients. In cases of PI-RADS 3 findings, individualized decision-making might be practical for patients and urologists if MRI quality and radiologists’ experience are high.

PSAD has emerged as a valuable parameter to enhance risk stratification in PC diagnostics, particularly in the MRI era. Compared to PSA alone, PSAD offers superior specificity for csPC as supported by recent studies and meta-analyses [13, 25–27]. In our cohort, PSAD values were predominantly elevated, with most patients presenting levels ≥ 0.15 ng/mL/cm³. Only 11% (32 men) had a PSAD below this threshold, and among them, just 9% were diagnosed with csPC. Notably, 73% of patients with negative biopsy results had a PSAD ≥ 0.15 ng/mL/cm³. These findings show limitations of PSAD in stratifying risk among high PSA populations. However, further research is needed to better define its utility in this subgroup, including the evaluation of adjusted cut-off values to enhance predictive accuracy and potentially reduce unnecessary biopsies. PSAD is mainly used as a biopsy trigger in populations with low or moderately elevated PSA levels (below 15 ng/mL). However, the extent to which PSAD is useful in collectives with significantly elevated PSA values remains questionable.

There are some limitations to be mentioned. First, the study was retrospective in nature, which inherently limits the ability to control confounding factors and introduces potential biases in patient selection and data completeness. PSA ≥ 15 ng/mL is not a commonly used cut-off for biopsy decision, but it is within an established intermediate-risk range used in multiple risk frameworks/trials and captures a cohort beyond the AS population. However, the choice of the defined cut-offs is debatable. Out of 376 total included consecutive patients with elevated PSA values ≥ 15 ng/mL, 96 did not undergo biopsy, or biopsy data were not available, and thus were not included in the further analysis. However, this could potentially have an influence on the quite high NPV (in comparison to the literature). Although follow-up data (with stable PSA and MRI findings) were collected, the absence of histopathological confirmation limits definitive conclusions on that. Moreover, long-term outcomes, including cancer progression or delayed detection of csPC, were not comprehensively assessed. The most critical limitation of this study indeed lies in the possibility of false-negative findings in both MRI and clinical follow-up. Although mpMRI has demonstrated high sensitivity for clinically significant PC, its negative predictive value is not absolute, and lesions (particularly small or low-grade PC) may remain occult. Similarly, clinical follow-up pathways are limited, and slowly growing or indolent cancers may evade detection for extended periods. While such PC may be of limited clinical relevance, their potential underrepresentation in our dataset must be acknowledged. While PSAD values were analyzed, no specific PSAD threshold was prospectively validated. Finally, we have no recorded detailed PSA-specific history, especially of medications or exercises with a potential influence on PSA values.

## Conclusions

In this high-risk cohort (PSA ≥ 15 ng/mL), mpMRI showed strong diagnostic performance for both detecting and excluding csPC. The high NPV for PI-RADS ≤ 3 supports its use as an effective triage tool to avoid unnecessary biopsies despite significantly elevated PSA levels. MpMRI maintained robust accuracy, reinforcing its value for individualized risk stratification and biopsy decision-making, suggesting that rigid PSA-based thresholds triggering invasive diagnostics should be re-evaluated.

## Data Availability

The datasets used and/or analyzed during the current study are available from the corresponding author on reasonable request.

## Abbreviations

csPC: Clinically significant prostate cancer
DCE: Dynamic contrast-enhanced
DWI: Diffusion-weighted imaging
ISUP GG: International Society of Urological Pathology Grade Group
mpMRI: Multiparametric magnetic resonance imaging
PC: Prostate cancer
PI-QUAL: Prostate imaging quality scoring system
PSA: Prostate-specific antigen
PSAD: Prostate-specific antigen density
TRUS: Transrectal ultrasound
